# ASFP (Artificial Intelligence based Scoring Function Platform): a web server for the development of customized scoring functions

**DOI:** 10.1186/s13321-021-00486-3

**Published:** 2021-02-04

**Authors:** Xujun Zhang, Chao Shen, Xueying Guo, Zhe Wang, Gaoqi Weng, Qing Ye, Gaoang Wang, Qiaojun He, Bo Yang, Dongsheng Cao, Tingjun Hou

**Affiliations:** 1 Hangzhou Institute of Innovative Medicine, College of Pharmaceutical Sciences, Zhejiang University, Hangzhou, Zhejiang 310058 China; 2 State Key Lab of CAD&CG, Zhejiang University, Hangzhou, Zhejiang 310058 China; 3 Xiangya School of Pharmaceutical Sciences, Central South University, Changsha, Hunan 10013 China

**Keywords:** Scoring functions, Descriptors, Machine learning, Virtual screening

## Abstract

Virtual screening (VS) based on molecular docking has emerged as one of the mainstream technologies of drug discovery due to its low cost and high efficiency. However, the scoring functions (SFs) implemented in most docking programs are not always accurate enough and how to improve their prediction accuracy is still a big challenge. Here, we propose an integrated platform called ASFP, a web server for the development of customized SFs for structure-based VS. There are three main modules in ASFP: (1) the descriptor generation module that can generate up to 3437 descriptors for the modelling of protein–ligand interactions; (2) the AI-based SF construction module that can establish target-specific SFs based on the pre-generated descriptors through three machine learning (ML) techniques; (3) the online prediction module that provides some well-constructed target-specific SFs for VS and an additional generic SF for binding affinity prediction. Our methodology has been validated on several benchmark datasets. The target-specific SFs can achieve an average ROC AUC of 0.973 towards 32 targets and the generic SF can achieve the Pearson correlation coefficient of 0.81 on the PDBbind version 2016 core set. To sum up, the ASFP server is a powerful tool for structure-based VS.

## Introduction

As one of the core technologies in virtual screening (VS), molecular docking has been extensively used to screen small molecule libraries for lead discovery [1]. A protein–ligand docking algorithm consists of two basic components: a search algorithm to generate a large number of potential ligand binding poses within the binding site and a scoring function (SF) to evaluate the binding strength for a particular pose. In general, most SFs implemented in docking programs cannot give a reliable prediction to the relative binding strength of a set of compounds [2]. Therefore, how to improve the accuracy of SFs still remains a big challenge.

In general, four parameters can be used to assess the prediction capability of a SF, including scoring power (binding affinity prediction), ranking power (relative ranking prediction), docking power (binding pose prediction), and screening power (discrimination of true binders from decoys) [3, 4]. In a VS campaign, the screening power of a SF is what we care about. Traditional SFs can be roughly classified into three categories: (1) force field-based SFs, (2) knowledge-based SFs and (3) empirical SFs. Unlike traditional SFs, machine learning (ML)-based scoring functions (MLSFs) do not have particular theory-motivated functional forms, and they are developed by learning from very large volumes of protein–ligand structural and interaction data through ML algorithms, such as random forest (RF), support vector machine (SVM), artificial neural network (ANN), gradient boosting decision tree (GBDT), etc [3,^ 5^–8]. Consequently, MLSFs have the capability to capture the non-linear relationship between protein–ligand interaction features and binding mode that are difficult to be characterized by classical SFs, thus yielding better binding strength predictions [9, 10]. However, in order to develop an MLSF, we need to generate a set of features to characterize protein–ligand interactions, and furthermore we need to be familiar with ML algorithms, which may be a difficult task for non-experts.

Here, we developed the ASFP server that can be used to develop customized MLSFs for structure-based VS and provide a generic MLSF for binding affinity prediction. The ASFP server has three basic modules: descriptor generation, AI-based SF construction and online prediction. In the descriptor generation module, 15 computational tools (only 9 tools are available due to license restriction) are embedded into the module for the characterization of ligand, protein binding pocket and protein–ligand interaction information, and up to 3437 descriptors can be generated. The AI-based SF construction module can be used to develop customized SFs with easy operation. In the online prediction module, 15 well-validated target-specific classification models for VS and an additional generic regression model for binding affinity prediction are provided for users. All the above modules in the ASFP server are automated and the results are presented interactively through a user-friendly interface.

## Implementation

The implementation of ASFP consists of two parts: the model construction and validation and the development of the web server that purposes in ML-based SF construction.

### Model construction

#### Benchmark

The benchmark dataset I (Dataset I), which contains the kinase subset and the diverse subset in the Directory of Useful Decoys-Enhanced (DUD-E) benchmark, was used to train and assess the MLSFs. The kinase subset contains the inhibitors and decoys generated by DUDE for 26 kinases, and the diverse subset contains the inhibitors and decoys for seven representative targets in the entire DUDE set. The basic information of Dataset I is shown in Additional file 1: Table S1.

The benchmark dataset II (Dataset II) extracted from the PDBbind database (version 2016) [11] was used to train and evaluate the SVM regression model for binding affinity prediction. There are 4057 protein–ligand complexes in the "refined set" and 290 complexes in the "core set" of PDBbind version 2016. The logarithm of one experimental measure from dissociation constant (K_d_), inhibition constant (K_i_) or concentration at 50% inhibition (IC_50_) was taken as the binding affinity of the protein–ligand complex (the priority is K_i_ > K_d_ > IC_50_ if two or three experimental measures are available for the target*)*.

#### Evaluation criteria

It should be noted that the target-specific models constructed in the ASFP server are classifiers used for the identification of binders from a pool of compounds (screening power) and the generic SF provided in the server is a regressor used for binding affinity prediction (scoring power). In this study, seven evaluation criteria were utilized to assess the performance of the models. Among them, F1 score, Cohen’s kappa, Matthews correlation coefficient (MCC), the area under the receiver operating characteristic curve (ROC AUC) were used to assess the classification performance of the target-specific SFs, and the enrichment factors (EF) at 0.5%, 1%, 2% and 5% were used to evaluate the early-recognition ability of target-specific models while the Pearson correlation coefficient (R_p_) and the root-mean-square error (RMSE) were calculated to assess the performance of the SVM regression model. The details of the metrics can be found in Additional file 1.

#### Preparation

The protein targets were prepared by using the *Structure Preparation wizard* in Schrodinger version 2018, which added hydrogen atoms, repaired the side-chains of the imperfect residues using Prime, and optimized the steric hindrance of side-chains. The protonation states of the proteins were determined by using PROPKA and the het groups were preprocessed by Epik to generate possible ionization and tautomeric states. The ligands were prepared using the *ligprep* module, which added hydrogen atoms, ionized the structures using Epik, desalted, generated tautomers and stereoisomers. In the preparation process, the default settings were used.

#### Docking

Two docking programs (i.e., Glide and Gold) were used for binding pose generation. When Glide was used for docking, the grids were firstly generated by using the *Receptor Grid Generation* utility with the size of binding box set to 10 Å × 10 Å × 10 Å centered on the co-crystallized ligand. Then, the Glide docking program with the SP scoring mode was used to dock the prepared ligands into the prepared proteins. For docking implemented by Gold, the binding site was defined by specifying the approximate center of the binding site and taking all atoms that lie within a 10 Å radius of this point, and ChemPLP was selected for scoring. For every ligand, only the pose with the highest docking score will be retained.

#### Descriptors generation

After molecular docking, the structural files of Dataset I and Dataset II were retained for descriptors generation. In this study, a total of 15 descriptors calculation tools of various types were included in computing descriptors (Table 1). Considering some of the tools were restricted by license, two schemes were employed to generate the descriptors to establish MLSFs. First, all the SFs (excluding fingerprints and dpocket) supported by the computational tools in Table 1 were used to generate descriptors (ALL descriptors). Second, all the SFs supported by the computational tools without licenses restrictions in Table 1 (i.e. AffiScore version 3.0, AutoDock version 6.8, DSX version 0.9, GalaxyDockBP2, NNScore version 2.01 and SMoG2016) were used to generate descriptors (FREE descriptors).

**Table 1:** The basic information of the computational tools supported by the descriptor generation module

Computational tools	Type of descriptors	No	Types
*AffiScore*^*1*^	Energy terms	13	Empirical
ASP^1^	Energy terms	5	Knowledge empirical
*AutoDock*	Energy terms	6	Force field
ChemPLP	Energy terms	11	Empirical
ChemScore	Energy terms	10	Empirical
*DPOCKET*	Pocket descriptors	49	–
*DSX*	Energy terms	1	Knowledge
*RDKit*	ECFP fingerprint	2048	–
*GalaxyDockBP2*	Energy terms	11	Empirical
Glide SP	Energy terms	17	Empirical
Glide XP	Energy terms	27	Empirical
GoldScore	Energy terms	6	Force field
*NNscore*	Energy terms	348	ML
*PaDEL*	Pubchem fingerprint	881	-
*SMoG2016*	Energy terms	5	Knowledge Empirical

^a^Computational tools without license restriction are marked in italics

#### Modeling

For the construction of target-specific MLSFs, the dataset for each target in Dataset I was split into the training set and test set with the ratio of 3:1, and preprocessed to scale the data and remove duplicated features. Then, three ML algorithms, including Support Vector Machine (SVM), Random Forest (RF) and eXtreme Gradient Boosting (XGboost), were used to develop the MLSF for each target, and the hyperparameters were optimized with the *hyperopt* package. During the hyper-parameter tuning process, the hyper-parameters were changed and then the model was assessed by a ten-fold cross-validation on the training set. The actual prediction performance of the final model with the optimal hyper-parameters was then assessed on the test set. To develop the generic SVM regression model for binding affinity prediction, the PDBbind version 2016 ‘refined set’ (excluding the PDBbind version 2016 ‘core set’) was used as the training set and the PDBbind version 2016 ‘core set’ was used as the test set.

### Web API

#### Descriptors generation

With respect to the characterization of protein–ligand interactions, energy terms and knowledge-based pairwise potentials extracted from existing SFs are popular representation methods. These energy components correlated with the binding affinity of protein–ligand complexes can be used as the input for the development of MLSFs. Therefore, 12 scoring programs were integrated into this module and the scoring components from the output of the SFs implemented in these computational tools can be generated automatically. Besides, two computational tools, i.e., RDkit and PaDEL, were integrated into this module to calculate the Extended-connectivity fingerprint (ECFP) and Pubchem fingerprint, respectively, to characterize the structural features of small molecules. Furthermore, the SF in dpocket was supported by this module to calculate 49 descriptors to characterize the structural information of protein pockets. It should be noted that the protein–ligand complexes should be docked before submitted to the server and the descriptors for small molecules may not be recommended for the development of MLSFs. The information of the 15 computational tools supported by ASFP are listed in Table 1. Because some computational tools implemented by ASFP are commercial, and therefore their functions are disabled. Based on the descriptors generated by this module, users can further construct a customized SF through a ML algorithm.

#### AI-based scoring functions construction

**Fig. 1:**
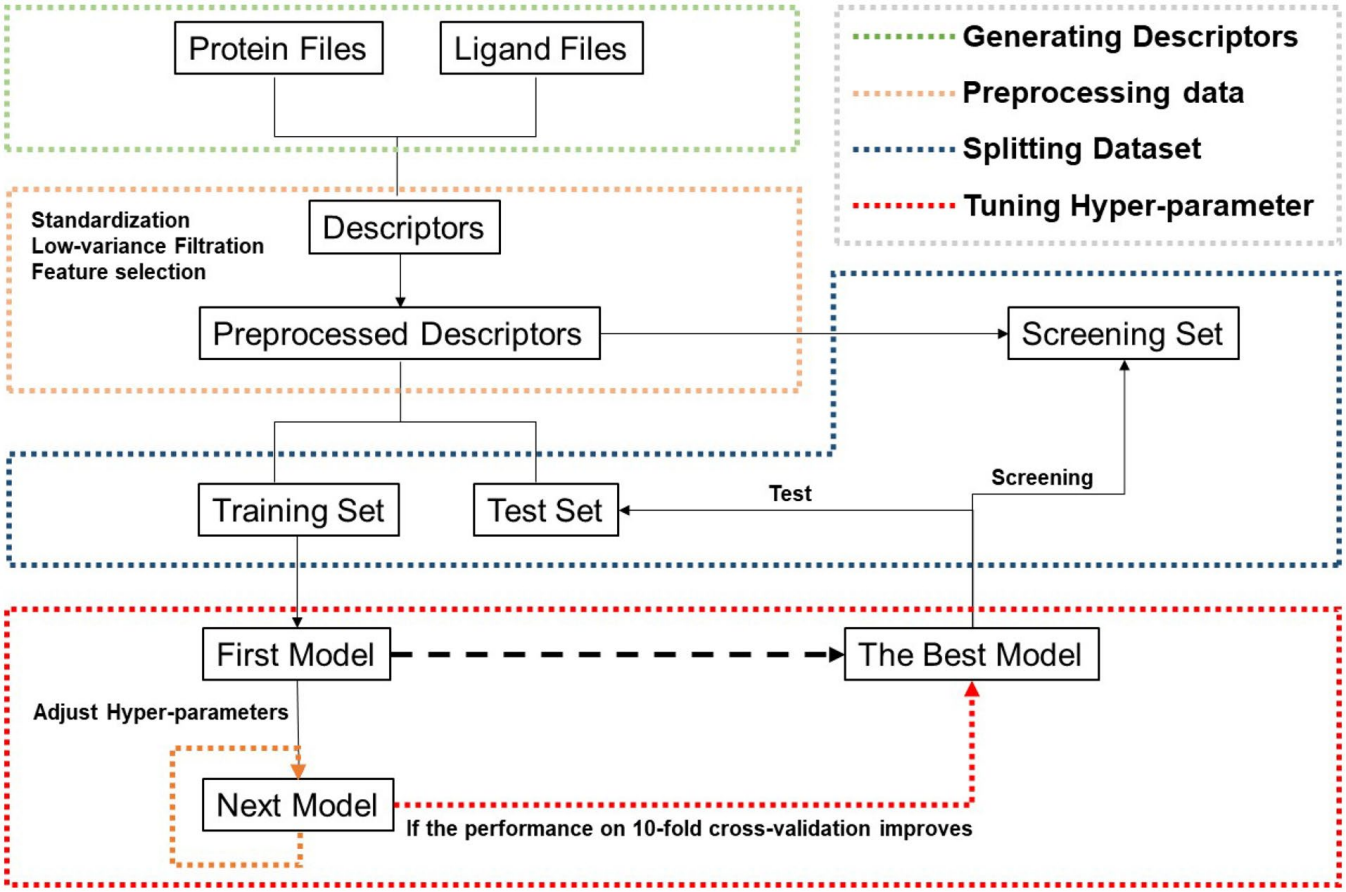
The workflow of the ASFP server for the AI-based scoring function construction

As one of the modules implemented in the server, the AI-based SF construction is designed for building customized target-specific MLSFs. After submission, the workflow is summarized in Fig. 1. In this module, the 384 descriptors computed and extracted from the SFs implemented in 6 freely available computational tools (AffiScore version 3.0, AutoDock version 6.8, DSX version 0.9, GalaxyDockBP2, NNScore version 2.01 and SMoG2016) can be used for training SFs. First, the whole dataset uploaded by the user is divided into the training set and the test set according to the user’s input. Then, the dataset is preprocessed (standardization, removing features with low variance, and tree-based feature selection) using *sklearn*. For the sake of computational efficiency, three popular ML algorithms (RF, SVM and XGBoost) are provided. Users can choose a ML algorithm for training and set some options about hyperparameter optimization (which hyperparameter to be optimized, the hyperparameter range and the optimization times). Finally, according to the user's input, the server uses *hyperopt* to find the optimal hyperparameter combinations and chooses the corresponding ML algorithm for training and prediction, and then outputs the results with a PDF file.

#### Online prediction

On the base of the model performance, 15 well-constructed customized SFs with research-worthy targets for VS and the generic regression SF for binding affinity prediction were retained to form the third module, Online prediction. The detailed information of the models is provided in Table 2.

**Table 2:** The information of the 15 targets with well-established classification models

Target	Data source	ML algorithm	95% confidence intervals for ROC_AUC on test set
abl1	DUD-E Kinase subset	SVM	0.969 ± 0.019
akt2			0.991 ± 0.014
csf1r			0.975 ± 0.017
egfr			0.986 ± 0.009
igf1r			0.963 ± 0.036
jak2			0.998 ± 0.002
kpcb			0.973 ± 0.023
mapk2			0.987 ± 0.009
mk01			0.963 ± 0.036
src			0.960 ± 0.019
tgfr1			0.994 ± 0.007
wee1			0.994 ± 0.011
akt1	DUD-E Diverse subset		0.987 ± 0.008
cxcr4			1.000 ± 0.000
hivpr			0.984 ± 0.009

The ASFP server based on a high-level Python web framework of Django is deployed on a Linux server of an Intel(R) Xeon(R) CPU E5-2630 v4 @ 2.20 GHz CPUs with 28 cores and 64 GB of memory. Several SFs programs like *autodock* [12] were integrated to automate the calculation process. The overall workflow implemented in the ASFP server is shown in Additional file 1: Figure S3, and the manual of ASFP can be downloaded from the website (http://cadd.zju.edu.cn/asfp/).

## Results

**Fig. 2:**
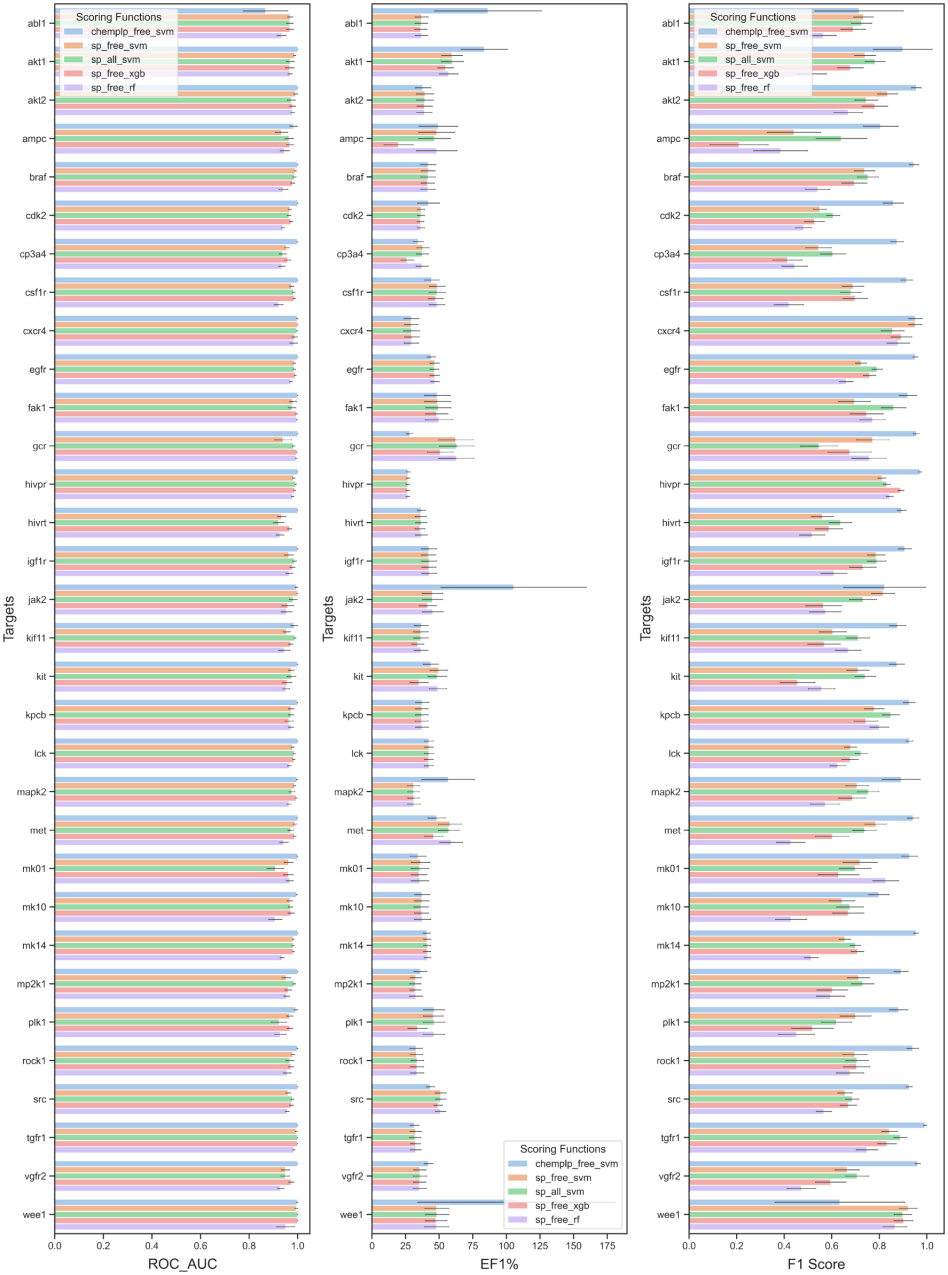
The performance of the customized SFs built by 3 ML algorithms (SVM, XGBoost and RF) and 2 traditional SFs (Glide SP and ChemPLP) on the Dataset I and their 95% confidence intervals by 10,000 bootstrapping for 3 metrics (ROC AUC, EF at 1% level and F1 Score). For the SF labels in this figure, ‘sp’ and ‘chemplp’ represent the docking methods (Glide SP and Gold ChemPLP) used for binding pose generation, ‘free’ and ‘all’ represent the descriptor combinations, and ‘svm’, ‘xgb’ and ‘rf’ are the ML algorithms used for modelling

**Fig. 3:**
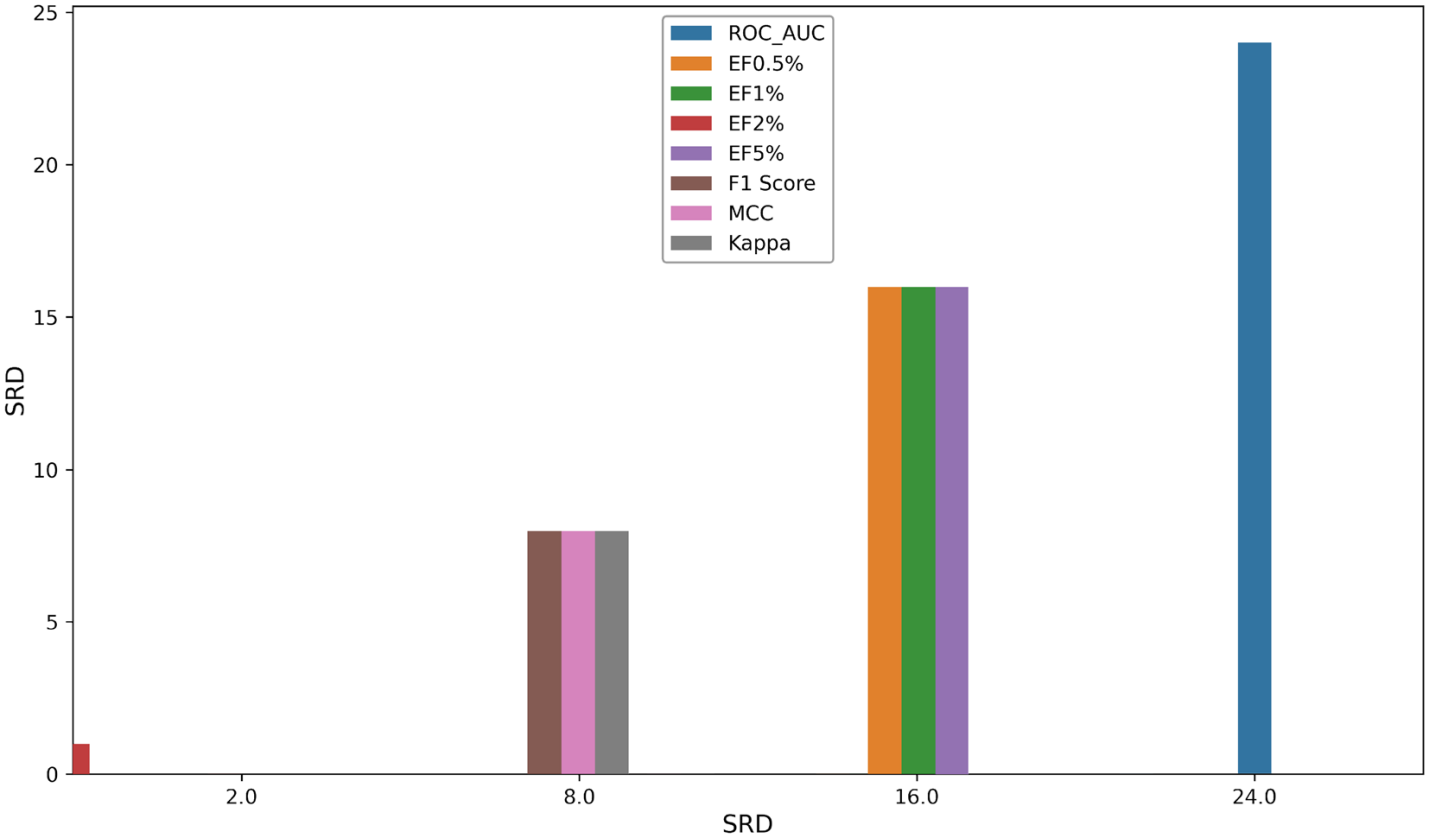
The results of the SRD analysis. SRD values are plotted on the X and left Y axes for visullization. (i) The models based on different algorithms are arranged in rows; (ii) a reference vector (row-wise data fusion, i.e., average) was defined and added as the last column of the matrix: this corresponds to an ideal reference method; (iii) the methods (columns) are ranked one-by-one in decreasing magnitude (including the reference column); (iv) the differences between the ranks of each sample between each method and the reference vector are calculated, and finally (v) these differences are summed for each method: these sums are called the SRD values, with the smaller value being the better (closer to the ideal reference method)

The performances of the customized SFs built by 3 ML algorithms (SVM, XGBoost and RF) and 2 traditional SFs (Glide SP and ChemPLP) on the Dataset I were assessed by 10,000 bootstrapping and the t-test for all the metrics (ROC AUC, EF0.5%, EF1%, EF2%, EF5%, F1 Score, MCC and Cohen’s kappa), and their 95% confidence intervals were calculated (Fig. 2 and Additional file 1: Figure S1-S2). The average performances of various SFs are listed in Table 2. All the seven metrics that can assess the quality of the ML SFs from different aspects may give conflicting rankings. Here, we implanted the sum of ranking differences (SRD) analysis (i.e., ranking the models by each metrics and the averages of all metrics respectively, and calculating the sum of the ranking differences between each metric and the averages) to compare the metrics to select better metrics for model comparison [13–15]. As shown in Fig. 3, the SRD scores of F1 Score, MCC and Cohen’s kappa are small, suggesting that the rankings based on them are similar to that based on the average metrics. Hence, the F1 Score was chosen for further model comparison.

As shown in Fig. 2 and Table 3, the target-specific SFs trained on the descriptors generated from the Glide poses outperformed the Glide SP SF (p < 0.05) and the models trained on the descriptors generated from the Gold poses outperformed the Gold ChemPLP SF (p < 0.05). Besides, the MLSFs constructed by ASFP outperformed the two tested docking methods, namely Glide and Gold, achieving an average ROC AUC of 0.973 towards 32 targets on the DUDE dataset. As for binding affinity prediction, the generic SF can achieve a Pearson correlation coefficient of 0.81 and a RMSE of 1.32 on the PDBbind version 2016 core set [11], highlighting its high prediction capability (Table 4). The average speed of modeling is 10 ligand per minute which is influenced by the ligand size and the computational capacities.

**Table 3:** Average performance of various SFs on Dataset I

Scoring functions	ROC_AUC	EF_0.5%_	EF_1%_	EF_2%_	EF_5%_	F1	MCC	Kappa
Glide@sp	0.634	10.386	7.779	5.289	3.353	–	–	–
Gold@chemplp	0.725	14.025	11.078	8.380	5.262	–	–	–
Dock	0.770	–	15.769	–	–	–	–	–
sp_free_svm	0.972	41.676	41.672	40.211	22.597	0.715	0.711	0.707
sp_free_xgb	0.977	41.147	38.353	26.178	10.940	0.661	0.692	0.655
sp_free_rf	0.955	41.743	41.618	38.099	17.828	0.607	0.604	0.598
sp_all_svm	0.972	41.607	41.583	40.486	21.924	0.731	0.728	0.724
chemplp_free_svm	0.993	53.625	46.801	41.027	21.272	0.897	0.897	0.894

The average performance of the customized SFs built by 3 ML algorithms (SVM, XGBoost and RF) in terms of 7 metrics (ROC AUC, EF at 0.5% level, EF at 1% level, EF at 2% level, EF at 5% level, F1 Score, MCC and Cohen’s kappa) and the performance of 2 traditional SFs (Glide SP and ChemPLP) in terms of 4 metrics (ROC AUC, EF at 0.5% level, EF at 1% level, EF at 2% level and EF at 5% level) on the Dataset I. For the SF labels in this figure, ‘sp’ and ‘chemplp’ represent the docking methods (Glide SP and Gold ChemPLP) used for binding pose generation, ‘free’ and ‘all’ represent the descriptor combinations, and ‘svm’, ‘xgb’ and ‘rf’ are the ML algorithms used for modelling

**Table 4:** The Scoring power of the regression SFs developed by the Online Prediction module

Model	Rp^a^	RMSE^b^
TopBP	0.861	1.65
TopBP-ML	0.848	1.74
TopBP-DL	0.848	1.64
ALL-SVM	0.831	1.23
EIC-Score	0.828	1.75
KDEEP	0.82	1.27
ΔvinaRF20	0.816	–
FREE-SVM	0.815	1.32
RI-Score	0.815	1.85
TNet-BP	0.81	1.34
Pafnucy	0.78	1.42
FFT-BP	0.747	–
X-Score	0.613	–

^a^Rp represents Pearson correlation coefficient (Rp)

^b^RMSE represents the root-mean-square error

To figure out the influence of various factors on model performance, the one-way ANOVA analyses were performed. First, we explored the impact of docking methods on model performance. The results illustrate that Gold ChemPLP performed better than Glide SP (p < 0.05) based on ROC AUC, and similarly the model built on the poses predicted by Gold outperformed that built on the poses generated by Glide, suggesting the binding poses generated by Gold may be closer to the true binding poses than those generated by Glide. Based on correct binding pose, our ASFP server can build reliable MLSFs for VS. Then, we studied the influence of different descriptor combinations on model performance (i.e., the descriptors generated from freely available and licensed software). Interestingly, the models built on the ALL descriptors outperform those trained on the FREE descriptors based on the F1 Score as expected (p < 0.05). It may be caused by more comprehensive characterization of protein–ligand interactions. Though the redundant descriptors that represent the same interactions between the descriptors generated by license-restricted SFs and FREE descriptors exist, the trick of the tree-based feature selection can offset the negative effect. Even so, the FREE descriptors used for modelling is also enough for building a target-specific SF with satisfactory screening power. We also compared the performance of three ML models implemented in our ASFP. The results illustrate that the ranking of average performance based on the F1 Score is SVM (F1 Score = 0.734), XGBoost (F1 Score = 0.690) and RF (F1 Score = 0.565) (p < 0.05). Therefore, SVM is the default option for modelling in the ASFP server. However, SVM also has its disadvantage of low calculation speed as it cannot compute in parallel. In that case, XGBoost and RF can be used as alternatives because they can be trained fast with acceptable performance.

## Discussion

All the three modules of ASFP required protein and ligand files uploaded and users can not only get satisfactory results as described in this paper by easily click the ‘Run’ button using default settings but also be allowed to submit jobs with their own settings. As mentioned in the previous section, the model performance relies on the quantity and quality of the training set and can be varied for different targets. Most of the 32 target-specific ML-based SFs constructed by ASFP outperform the classic SF (Glide SP and Gold) and can be built easily through the ASFP server. Therefore, our ASFP server is a powerful tool that can calculate descriptors for modeling and construct ML-based SFs for VS.

**Fig. 4:**
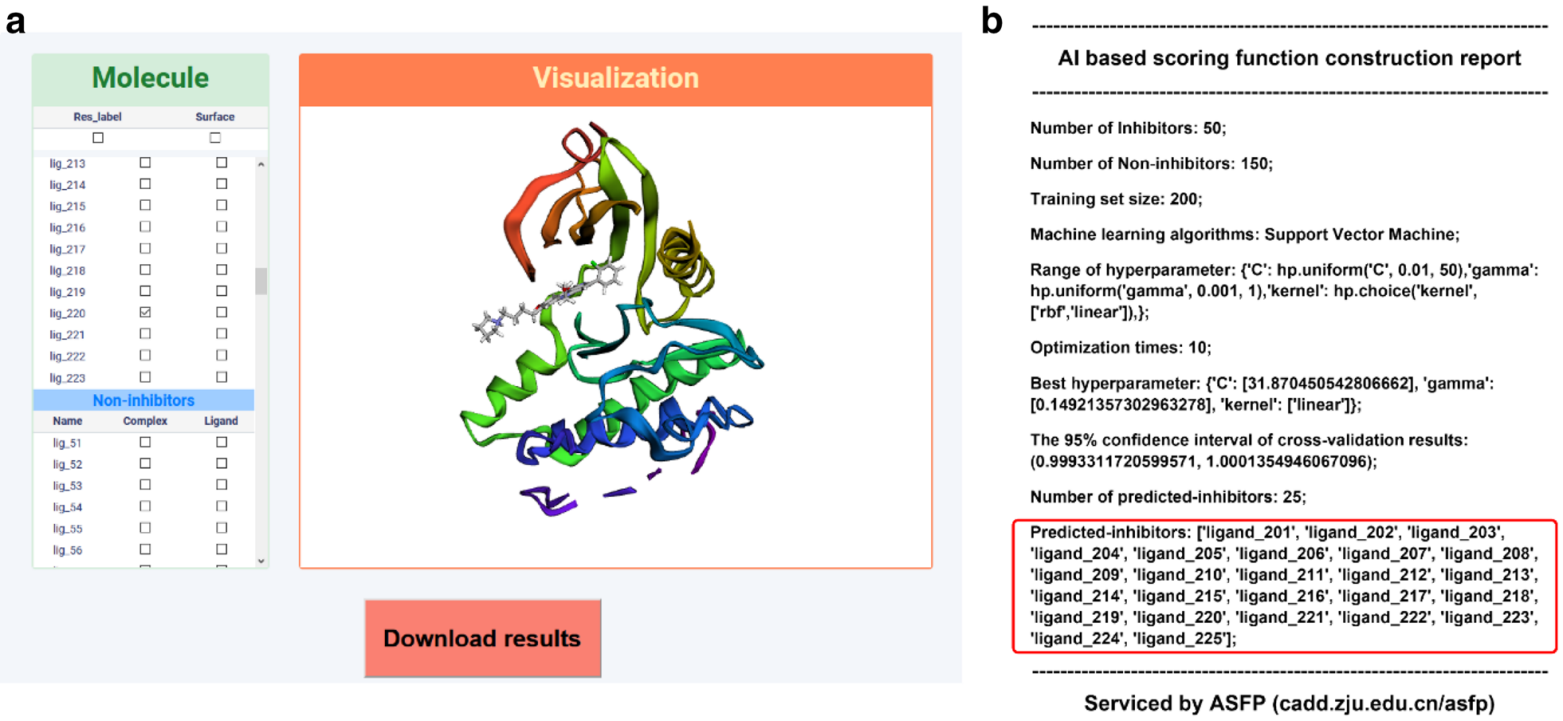
The AI-based scoring function construction result of the example (target: abl1). **a** The Visualization page of the results. **b** The prediction results in the report PDF file. The F1 score is reported in the "Cross validation results" section of the report

To illustrate the practicability of the ASFP server, if one wants to construct an ML-based SF to find ligands targeting at Tyrosine-protein kinase ABL (abl1), one can use the AI-Based Scoring Functions Construction module with the input files including a ligand file in the MOL2 format containing 50 active molecules, a decoy file in the MOL2 format containing 150 molecules, a test file in the MOL2 format containing 100 molecules and a protein file in the PDB format (PDB ID: 2HZI [16]). Upload the files and submit the job with the default hyperparameters settings. As shown in Fig. 4, the ASFP server succeeds in generating descriptors and constructing a customized MLSF. The returned PDF file shows that the SF successfully identifies 25 inhibitors from 100 molecules (25 inhibitors).

## Conclusions

Here, we present a user-friendly ASFP server for customizing SFs for structure-based VS. We have validated our methodology on several benchmark datasets, and the target-specific SFs constructed by ASFP achieved an average ROC AUC of 0.973 towards 32 targets on the DUDE dataset and the generic SF can achieve the Pearson correlation coefficient of 0.81 on the PDBbind version 2016 core set, suggesting that the ASFP server is a useful and effective tool for MLSF construction. The combination of 15 computational descriptor generation tools, *sklearn* and *hyperopt* makes it very convenient to calculate different types of descriptors and construct customized MLSFs. The ASFP server is an on-going project and further developments will be focused on the integration of more descriptor generation tools, the development of an automatic modelling pipeline using deep learning algorithms (e.g. 3D-convolutional neural networks) and the acceleration in computational speed with the help of more computing resources.

## Availability and requirements

- Project name: ASFP (Artificial Intelligence based Scoring Function Platform)
- Project home page: http://cadd.zju.edu.cn/asfp/
- Operating system(s): Platform independent
- Programming language: Python
- Other requirements: Mozilla Firefox or Google Chrome is recommended
- License: MIT
- Any restrictions to use by non-academics: no

## Supplementary Information

**Additional file 1. **Supplementary materials.

## Data Availability

The web server is available at http://cadd.zju.edu.cn/asfp/. The ASFP manual is available at http://cadd.zju.edu.cn/asfp/extract/download/?name=h. The data and source code are available at https://github.com/5AGE-zhang/ASFP. ASFP web server is freely available at http://cadd.zju.edu.cn/asfp/.
