# Supplementary material for: ASFP (Artificial Intelligence based Scoring Function Platform): a web server for the development of customized scoring functions
Source: J Cheminform. 2021 Feb 4;13:6. doi: 10.1186/s13321-021-00486-3 (PMC7860246; doi:10.1186/s13321-021-00486-3)
Supplement: Supplementary file 1 — Additional file 1. Supplementary materials. [file 13321_2021_486_MOESM1_ESM.docx]

**Supplementary Materials**

**Part I: The process to train the MLSFs**

**1.1. Benchmark datasets**

The benchmark dataset I (Dataset I), which contains the kinase subset and the diverse subset in the Directory of Useful Decoys-Enhanced (DUD-E) benchmark, was used to train and assess the MLSFs^1^. The kinase subset contains the inhibitors and decoys generated by DUDE for 26 kinases, and the diverse subset contains the inhibitors and decoys for seven representative targets in the entire DUDE set. The basic information of Dataset I is shown in Table S1.

The benchmark dataset II (Dataset II) extracted from the PDBbind database (version 2016) was used to train and evaluate the SVM regression model for binding affinity prediction^2^. There are 4057 protein-ligand complexes in the "refined set" and 290 complexes in the "core set" of PDBbind version 2016.

**Table S1**. Detailed information of the entries in Dataset I.

| Subset | Target | PDB code | Actives number | Decoys number |
| --- | --- | --- | --- | --- |
| Kinase | ABL1 | 2hzi | 278 | 10402 |
|  | AKT1 | 3cqw | 247 | 14340 |
|  | AKT2 | 3d0e | 184 | 6840 |
|  | BRAF | [3d4q](http://www.rcsb.org/pdb/explore/explore.do?structureId=3d4q) | 246 | 9924 |
|  | [CDK2](http://dude.docking.org/targets/cdk2) | [1h00](http://www.rcsb.org/pdb/explore/explore.do?structureId=1h00) | 798 | 28158 |
|  | [CSF1R](http://dude.docking.org/targets/csf1r) | [3krj](http://www.rcsb.org/pdb/explore/explore.do?structureId=3krj) | 265 | 12383 |
|  | [EGFR](http://dude.docking.org/targets/egfr) | [2rgp](http://www.rcsb.org/pdb/explore/explore.do?structureId=2rgp) | 765 | 34735 |
|  | [FAK1](http://dude.docking.org/targets/fak1) | [3bz3](http://www.rcsb.org/pdb/explore/explore.do?structureId=3bz3) | 115 | 5380 |
|  | [IGF1R](http://dude.docking.org/targets/igf1r) | [2oj9](http://www.rcsb.org/pdb/explore/explore.do?structureId=2oj9) | 227 | 9323 |
|  | [JAK2](http://dude.docking.org/targets/jak2) | [3lpb](http://www.rcsb.org/pdb/explore/explore.do?structureId=3lpb) | 154 | 6525 |
|  | [KIT](http://dude.docking.org/targets/kit) | [3g0e](http://www.rcsb.org/pdb/explore/explore.do?structureId=3g0e) | 221 | 10334 |
|  | [KPCB](http://dude.docking.org/targets/kpcb) | [2i0e](http://www.rcsb.org/pdb/explore/explore.do?structureId=2i0e) | 248 | 8808 |
|  | [LCK](http://dude.docking.org/targets/lck) | [2of2](http://www.rcsb.org/pdb/explore/explore.do?structureId=2of2) | 675 | 27753 |
|  | [MAPK2](http://dude.docking.org/targets/mapk2) | [3m2w](http://www.rcsb.org/pdb/explore/explore.do?structureId=3m2w) | 207 | 6167 |
|  | [MET](http://dude.docking.org/targets/met) | [3lq8](http://www.rcsb.org/pdb/explore/explore.do?structureId=3lq8) | 198 | 11047 |
|  | [MK01](http://dude.docking.org/targets/mk01) | [2ojg](http://www.rcsb.org/pdb/explore/explore.do?structureId=2ojg) | 129 | 4325 |
|  | [MK10](http://dude.docking.org/targets/mk10) | [2zdt](http://www.rcsb.org/pdb/explore/explore.do?structureId=2zdt) | 187 | 6606 |
|  | [MK14](http://dude.docking.org/targets/mk14) | [2qd9](http://www.rcsb.org/pdb/explore/explore.do?structureId=2qd9) | 910 | 36288 |
|  | [MP2K1](http://dude.docking.org/targets/mp2k1) | [3eqh](http://www.rcsb.org/pdb/explore/explore.do?structureId=3eqh) | 218 | 6687 |
|  | [PLK1](http://dude.docking.org/targets/plk1) | [2owb](http://www.rcsb.org/pdb/explore/explore.do?structureId=2owb) | 155 | 6853 |
|  | [ROCK1](http://dude.docking.org/targets/rock1) | [2etr](http://www.rcsb.org/pdb/explore/explore.do?structureId=2etr) | 202 | 6351 |
|  | [SRC](http://dude.docking.org/targets/src) | [3el8](http://www.rcsb.org/pdb/explore/explore.do?structureId=3el8) | 653 | 32436 |
|  | [TGFR1](http://dude.docking.org/targets/tgfr1) | [3hmm](http://www.rcsb.org/pdb/explore/explore.do?structureId=3hmm) | 260 | 8040 |
|  | [VGFR2](http://dude.docking.org/targets/vgfr2) | [2p2i](http://www.rcsb.org/pdb/explore/explore.do?structureId=2p2i) | 225 | 7629 |
|  | [WEE1](http://dude.docking.org/targets/wee1) | [3biz](http://www.rcsb.org/pdb/explore/explore.do?structureId=3biz) | 138 | 6213 |
| Diverse | AMPC | 1l2s | 63 | 2786 |
|  | CP3A4 | 3nxu | 294 | 10465 |
|  | CXCR4 | 3odu | 123 | 3403 |
|  | GCR | 3bqd | 99 | 6001 |
|  | HIVPR | 1xl2 | 1396 | 36278 |
|  | HIVRT | 3lan | 315 | 11150 |
|  | KIF11 | 3cjo | 198 | 6910 |

**1.2. Molecular docking and development of MLSFs**

*Preparation*. The protein targets were prepared by using the *Structure Preparation wizard* in Schrodinger version 2018^3^, which added hydrogen atoms, repaired the side-chains of the imperfect residues using Prime^4^, and optimized the steric hindrance of side-chains. The protonation states of the proteins were determined by using PROPKA and the het groups were preprocessed by Epik to generate possible ionization and tautomeric states.^5^ The ligands were prepared using the *ligprep* module, which added hydrogen atoms, ionized the structures using Epik, desalted, generated tautomers and stereoisomers. In the preparation process, the default settings were used.

*Docking.* Two docking programs (i.e. Glide and Gold) were used for binding pose generation. When Glide was used for docking, the grids were firstly generated by using the Receptor Grid Generation utility with the size of binding box set to 10 Å × 10 Å × 10 Å centered on the co-crystallized ligand. Then, the Glide docking program with the SP scoring mode was used to dock the prepared ligands into the prepared proteins. For docking implemented by Gold, the binding site was defined by specifying the approximate center of the binding site and taking all atoms that lie within a 10 Å radius of this point and ChemPLP was selected for scoring. For every ligand, only the pose with the highest docking score will be retained.

*Descriptor generation*. After molecular docking, the structural files of Dataset I and Dataset II were uploaded to the ASFP server for descriptor generation. Because some computational tools implemented by ASFP are commercial, and therefore their functions are disabled. Two schemes were employed to generate the descriptors to establish MLSFs. First, all the SFs (excluding fingerprints and dpocket) supported by the computational tools in Table 1 were used to generate descriptors (ALL descriptors). Second, all the SFs supported by the computational tools without licenses restrictions in Table 1 were used to generate descriptors (FREE descriptors).

*Development of MLSFs*. For the construction of target-specific MLSFs, the dataset for each target in Dataset I was split into the training set and test set with the ratio of 3:1, and preprocessed to scale the data and remove duplicated features. Then, three ML algorithms, including Support Vector Machine (SVM)^6^, Random Forest (RF)^7^ and eXtreme Gradient Boosting (XGboost)^8^, were used to develop the MLSF for each target, and the hyperparameters were optimized with the *hyperopt* package. During the process of hyper-parameters’ tuning, the hyper-parameters were changed and then the model was assessed by a ten-fold cross-validation on the training set. The actual prediction performance of final model with optimal hyper-parameters was then assessed on the test set. To develop the generic SVM regression model for binding affinity prediction, the PDBbind version 2016 ‘refined set’ (excluding the PDBbind version 2016 ‘core set’) was used as the training set and the PDBbind version 2016 ‘core set’ was used as the test set.

**1.3. Evaluation criteria**

In this study, seven evaluation criteria were utilized to assess the performance of the models. Among them, F1 score, Cohen’s kappa, Matthews correlation coefficient (MCC), the area under the receiver operating characteristic curve (ROC AUC) and the enrichment factor (EF) at 0.5%, 1%, 2% and 5% were used to evaluate the performance of target-specific models while the Pearson correlation coefficient (*R*_p_) and the root-mean-square error (RMSE) were calculated to assess the performance of the SVM regression model.

F1 score can be interpreted as a weighted average of the precision and recall, where an F1 score reaches its best value of 1 and the worst score of 0. Cohen’s kappa is a score that expresses the level of agreement between two annotators on a classification problem. MCC is used as a measure of the quality of binary and multiclass classifications, and 1 represents a perfect prediction, 0 an average random prediction and -1 an inverse prediction. ROC AUC illustrates the performance of a binary classifier of which the range is between 0 and 1. AUC=1 means a perfect discrimination and AUC=0.5 means a random discrimination. However, in practice, only tens to hundreds of top-ranked molecules will be experimentally tested^9^, and therefore, EF is used here as a measure of the capability of a MLSF to identify active molecules and it represents the ratio between the proportion of actives at the top x% molecules and the proportion of actives in the entire dataset (EF_x%_). The *R*_p_ is widely used to measure the degree of correlation between two variables, and its value is between -1 and 1. The root-mean-square error (RMSE) is a frequently used measure of the differences between values (sample or population values) predicted by a model or an estimator and the values observed. RMSE is always non-negative, and a value of 0 (almost never achieved in practice) would indicate a perfect fit to the data.

**1.4. Model performance**

The performance of the customized SFs built by the ASFP server are shown in Figure S1 and Figure S2.


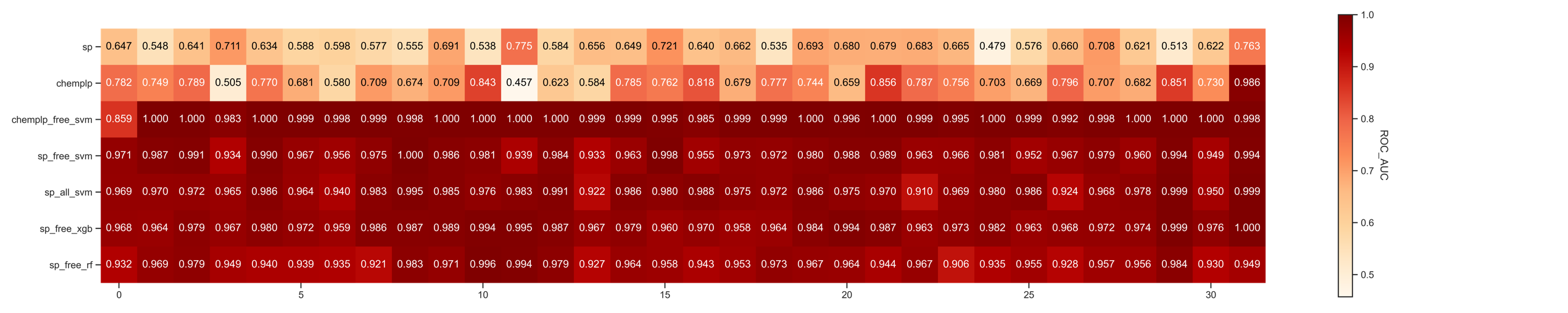


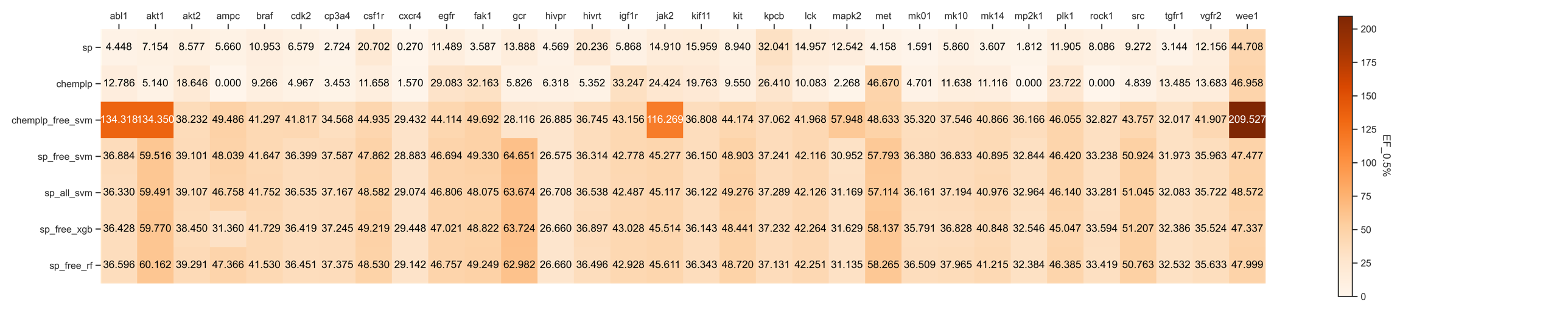

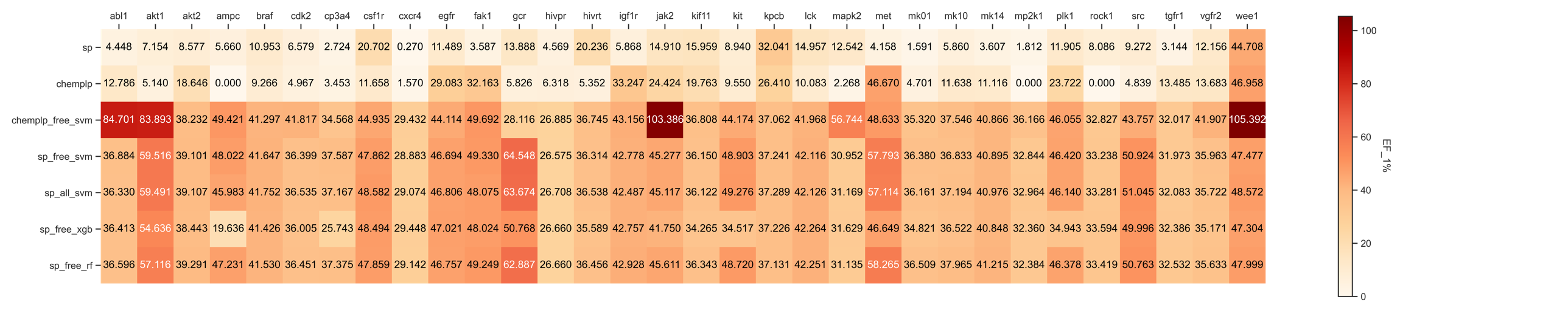

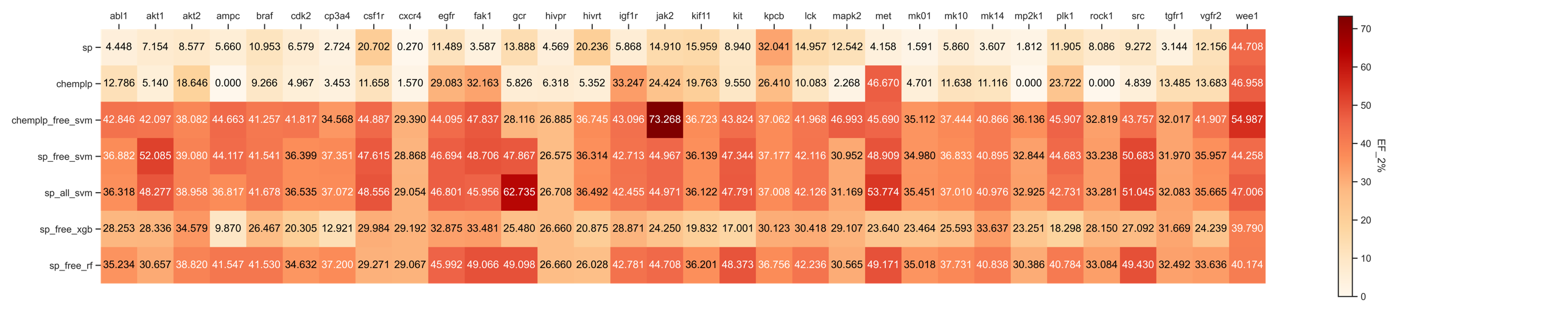

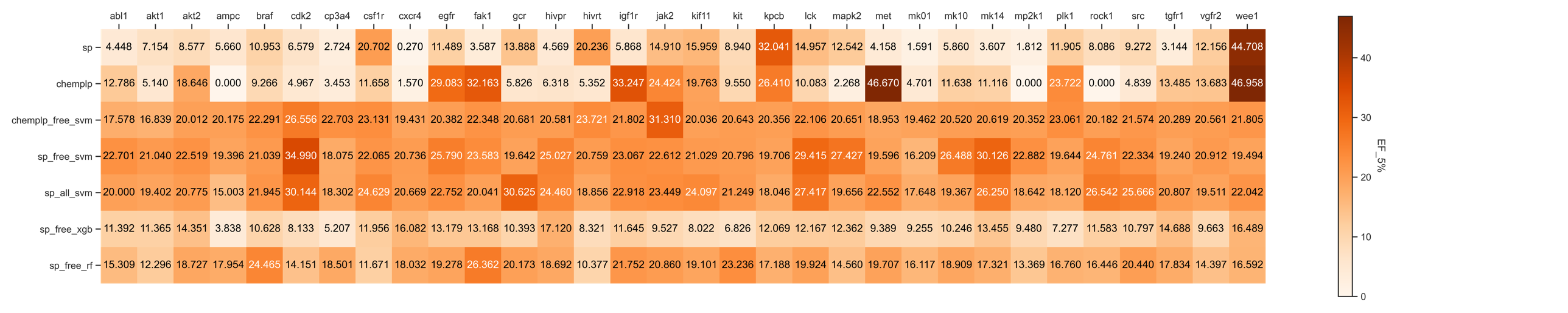

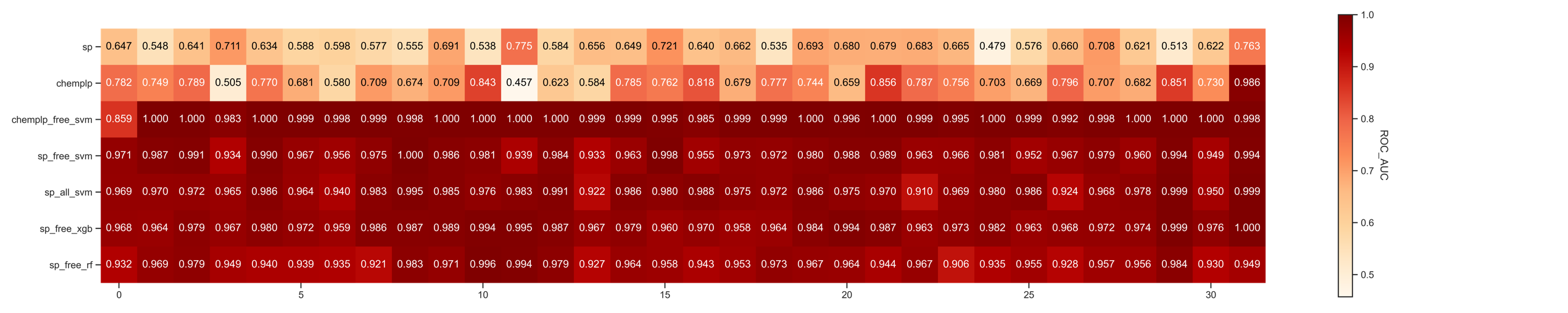

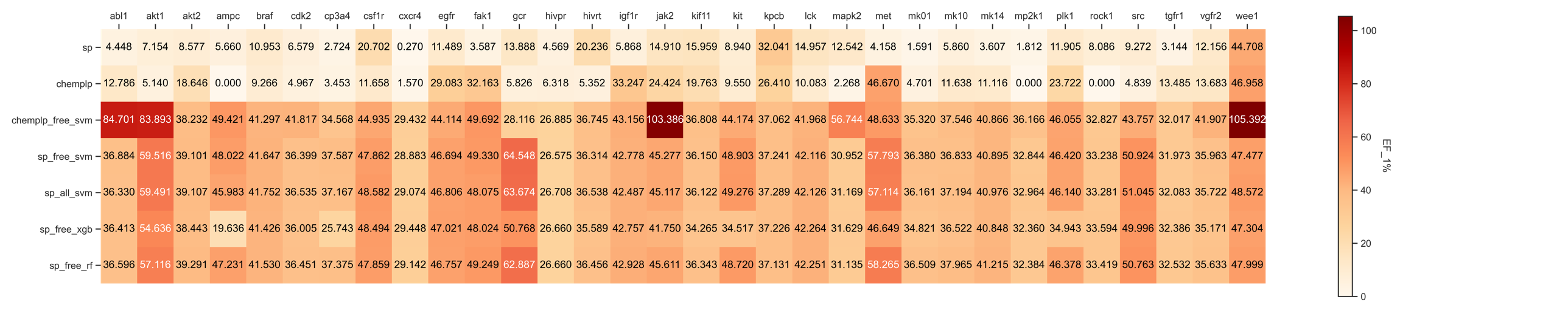

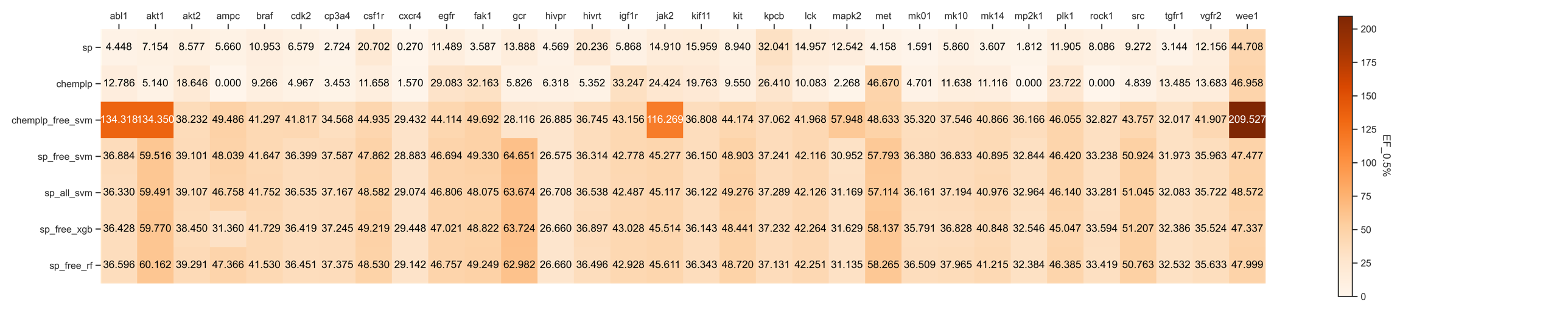


**Figure S1.** The performance of the customized SFs built by 3 ML algorithms (SVM, XGBoost and RF) in terms of 7 metrics (ROC AUC, EF at 0.5 % level, EF at 1 % level, EF at 2% level, EF at 5 % level, F1 Score, MCC and Cohen’s kappa) and the performance of 2 traditional SFs (Glide SP and ChemPLP) in terms of 4 metrics (ROC AUC, EF at 0.5 % level, EF at 1 % level, EF at 2% level and EF at 5 % level) on the Dataset I. For the SF labels in this figure, ‘sp’ and ‘chemplp’ represent the docking methods (Glide SP and Gold ChemPLP) used for binding pose generation, ‘free’ and ‘all’ represent the descriptor combinations, and ‘svm’, ‘xgb’ and ‘rf’ are the ML algorithms used for modelling.







**Figure S2.** The performance of the customized SFs built by 3 ML algorithms (SVM, XGBoost and RF) and 2 traditional SFs (Glide SP and ChemPLP) on the Dataset I and their 95 % confidence intervals by 10000 bootstrapping for 5 metrics (EF at 0.5 % level, EF at 2% level, EF at 5 % level, MCC and Cohen’s kappa). For the SF labels in this figure, ‘sp’ and ‘chemplp’ represent the docking methods (Glide SP and Gold ChemPLP) used for binding pose generation, ‘free’ and ‘all’ represent the descriptor combinations, and ‘svm’, ‘xgb’ and ‘rf’ are the ML algorithms used for modelling.

**Part 2: The development of the ASFP server**

**2.1. Web server** **implementation**

The ASFP server is an integrated web server that combines the calculation of scoring components extracted from traditional SFs, molecular fingerprints, and protein pocket descriptors for descriptor generation and *sklearn* and *hyperopt* for MLSF construction. The ASFP pipeline is shown in Figure S3 and the descriptor generation tools are listed in Table S3. The ASFP server based on a high-level Python web framework of Django is deployed on a Linux server of an Intel(R) Xeon(R) CPU E5-2630 v4 @ 2.20GHz CPUs with 28 cores and 64 GB of memory. After submitting the jobs, the ASFP server will create the corresponding tasks and then put them into the queue immediately. A job of descriptor generation under the energy terms mode provided by ASFP reaches a speed at about 10 ligands per minute, which is also influenced by the ligands’ size. Under the other modes the calculation speed depends on the tools chosen by users. The speed of an AI-based SF construction or online prediction job is usually a little slower than 10 ligands per minute. Afterwards, the web interface will return a PDF file containing information about users’ input and three Uniform Resource Locators (URLs) for checking job statuses and downloading results. One of the URLs leads to the Queue page on which users are allowed to check their jobs’ status. Besides, the URL of the results Visualization page is also saved in the PDF file and if the calculation is not finished, the same URL will be directed to a WAIT page. The URL of the page is unique for each job and therefore users should save the PDF file properly.

**Table S3**. Descriptor generation tools implemented in ASFP.

| Descriptor type | Energy terms | | Fingerprints | Pockets |
| --- | --- | --- | --- | --- |
| Number | 12 | | 2 | 1 |
| Name | AffiScore  ASP^10^  AutoDock  ChemPLP  ChemScore  DSX | GalaxyDockBP2  Glide sp^11-14^  Glide xp  GoldScore  NNscore  SMoG2016 | RDKit  PaDEL | DPOCKET |


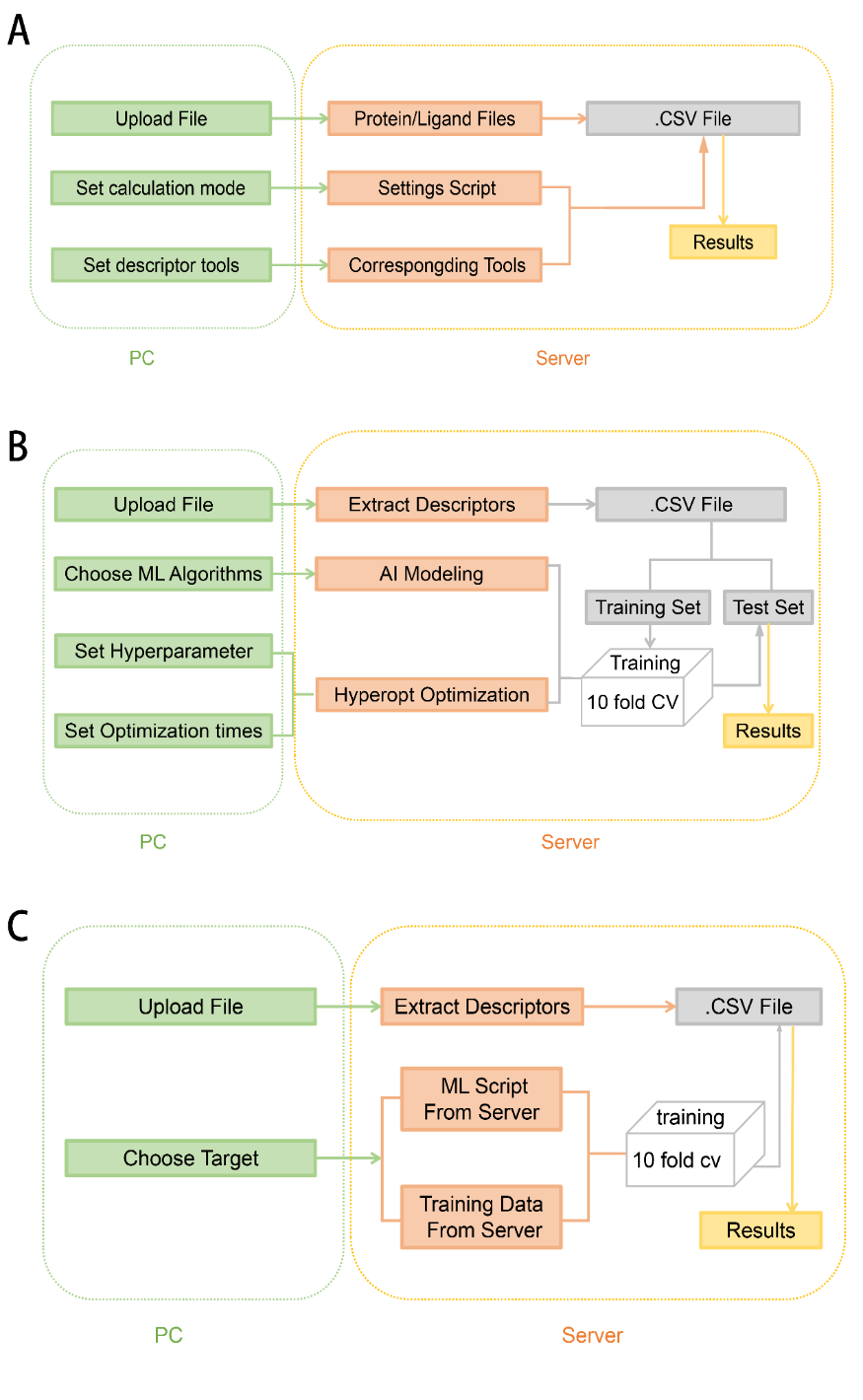


**Figure S3**. The workflow of the ASFP server for (A) the descriptor generation module, (B) the AI-based scoring function construction module, and (C) the online prediction module.

**2.2. Input**

The functions of the ASFP server include generating a number of descriptors that characterize protein-ligand interactions, constructing customized MLSFs with one of the three ML algorithms and utilizing 15 accurate classification models for VS or the ML regression model for binding affinity prediction.

Figure S4A shows the user interface of the descriptor generation module. In this module, users are requested to input a string with letters, numbers and/or underlines as their unique jobname. Besides, a ZIP file is needed for the calculation of descriptors. For the target-specific (one protein and multiple ligands) descriptor generation, the ZIP file should contain a protein file (PDB) and a ligand file (SDF/MOL2). For the generic (several protein-ligand complexes) descriptor calculation, the ZIP file should contain the same number of folders as the target number, and each folder needs to contain a protein file and a ligand file (one molecule). The ASFP server will determine whether to calculate the target-specific descriptors or the generic descriptors according to the uploaded ZIP file. Once users have finished the operations mentioned above, they have to decide to select which descriptor generation tools. There are two ways to choose descriptor generation tools: 1) choose directly from the table on the right of the web page and 2) quickly select a series of descriptor generation tools using the buttons provided by ASFP. When the jobs are finished, the users will get a PDF file containing the information of their input and the URL related to the result immediately.

Figure S4B shows the user interface of the AI-based SF construction module. In this module, the job still needs a name with the same required by the descriptor generation module. However, the requirement of the input files is different from that for the descriptor generation module. Users using this module are asked to upload a protein file in the PDB format whose size should be larger than 20 kb, a ligand file in the MOL2 or SDF format in which the molecules should be binders to the protein, a decoy file in the MOL2 or SDF format in which the molecules should be nonbinders to the protein, and a test file in the MOL2 or SDF format for prediction. After uploading the required files, users should choose a ML algorithm from the three ML algorithms (SVM, RF and XGboost) provided by ASFP. It is well-known that tuning hyperparameters is of great importance to the prediction performance of a ML model^15-19^. If users are familiar with *sklearn*, they can change the default settings of hyperparameters by easily selecting the options on the selection box or Minimum and Maxum in the Min Input box and Max input box, respectively, to decide which hyperparameters to be optimized and the range of these hyperparameters. Moreover, users are allowed to input a number to decide the optimization times of hyperparameters if they want to. Similar to the descriptor generation module, once users submit their jobs, they will get a PDF file immediately to summarize the information of the input and URLs related to the calculation results.

Figure S4C shows the user interface of the online prediction module. The requirement of the jobname and input files are exactly the same as those for the descriptor generation module. The ASFP server will determine whether to call the target-specific model or the generic model according to the uploaded ZIP file. As to the prediction using a target-specific model, users should choose the corresponding model. As to the binding affinity prediction using the generic model, users do no need to choose any model. Once users submit their jobs, they will also get a PDF file of the results.

***
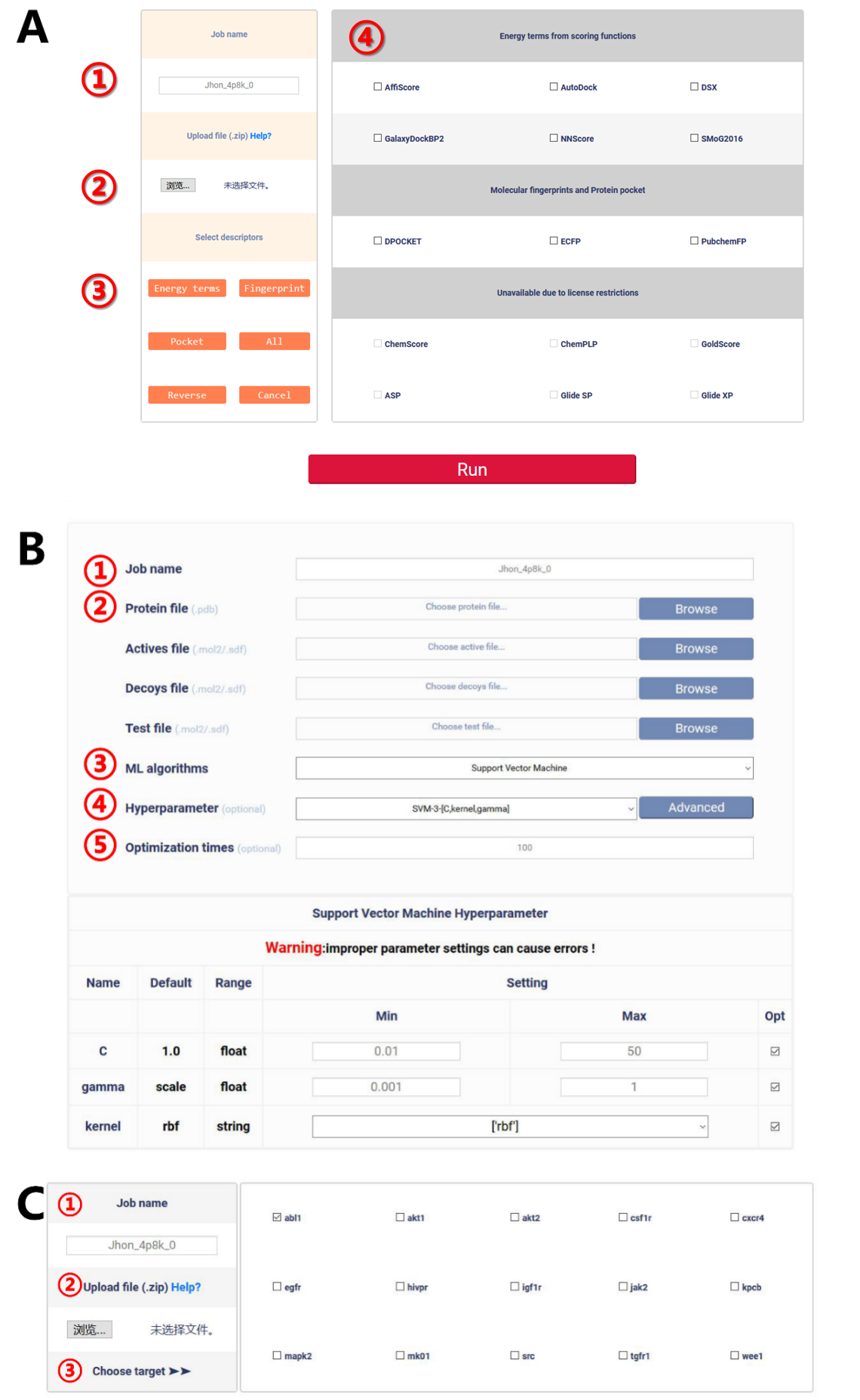
***

**Figure S4**. Data inputs in the ASFP server. (A) A descriptor generation job needs: (1) a job name, (2) a protein file in the PDB format and a ligand file in the SDF or MOL2 format, (3) the selection of descriptor calculation tools, (4) descriptor generation tools. (B) An AI-based scoring function construction job needs: (1) a job name, (2) a protein file in the PDB format and a ligand file in the SDF or MOL2 format, (3) the selection of a ML algorithm, and (4 and 5) settings of the hyperparameters’ optimization. (C) An online prediction job needs: (1) a job name, (2) a protein file in the PDB format and a ligand file in the SDF or MOL2 format, and (3) the selection of the corresponding target (for the SVM regression model, no option is needed).

**2.3. Output**

Once a submitted job is finished, the WAIT page will be auto-refreshed to the result Visualization page or users can click the URL in the PDF file to the Visualization page. As shown in Figure S5, the format and contents of the result depend on the job types.

Figure S5A shows the five components of the Visualization page. The main function of this page for users is to illustrate the protein-ligand binding structure with the support of 3Dmol.js, a WebGL-based molecular viewer^20^. Except the URL provided in the PDF file, users can download the results directly by easily clicking the Download button on the Visualization page. For the results of the AI scoring function construction and online prediction, the ligand checkboxes will be shown in two tables, named Inhibitors and Non-inhibitors, while for the results of the descriptor generation, the ligand checkboxes are shown in one box. In addition, for the results of a target-specific job, users can select multiple checkboxes at the same time to compare the differences of their binding modes.

Figure S5B shows different result files of different modules from ASFP. PDF and CSV are the two main formats of the results and all the result files are compressed into a ZIP file with the same name as the job name. For the descriptor generation module, the results are CSV files named in tools’ name containing the descriptors calculated using the corresponding tools. For the AI SF construction and online prediction module, users will get a ZIP file that contains the descriptor files in the CSV format and a prediction report file in the PDF format. The prediction report file consists of four parts: 1) information about the training set and information about the test set for the online prediction module; 2) information about the ML algorithm for modelling and its hyperparameters; 3) model performance on the test set for the online prediction module and the values of the ten-fold cross validation’s AUC to assess the performance of the model for the AI SF construction; 4) the prediction result is shown in a list.

***
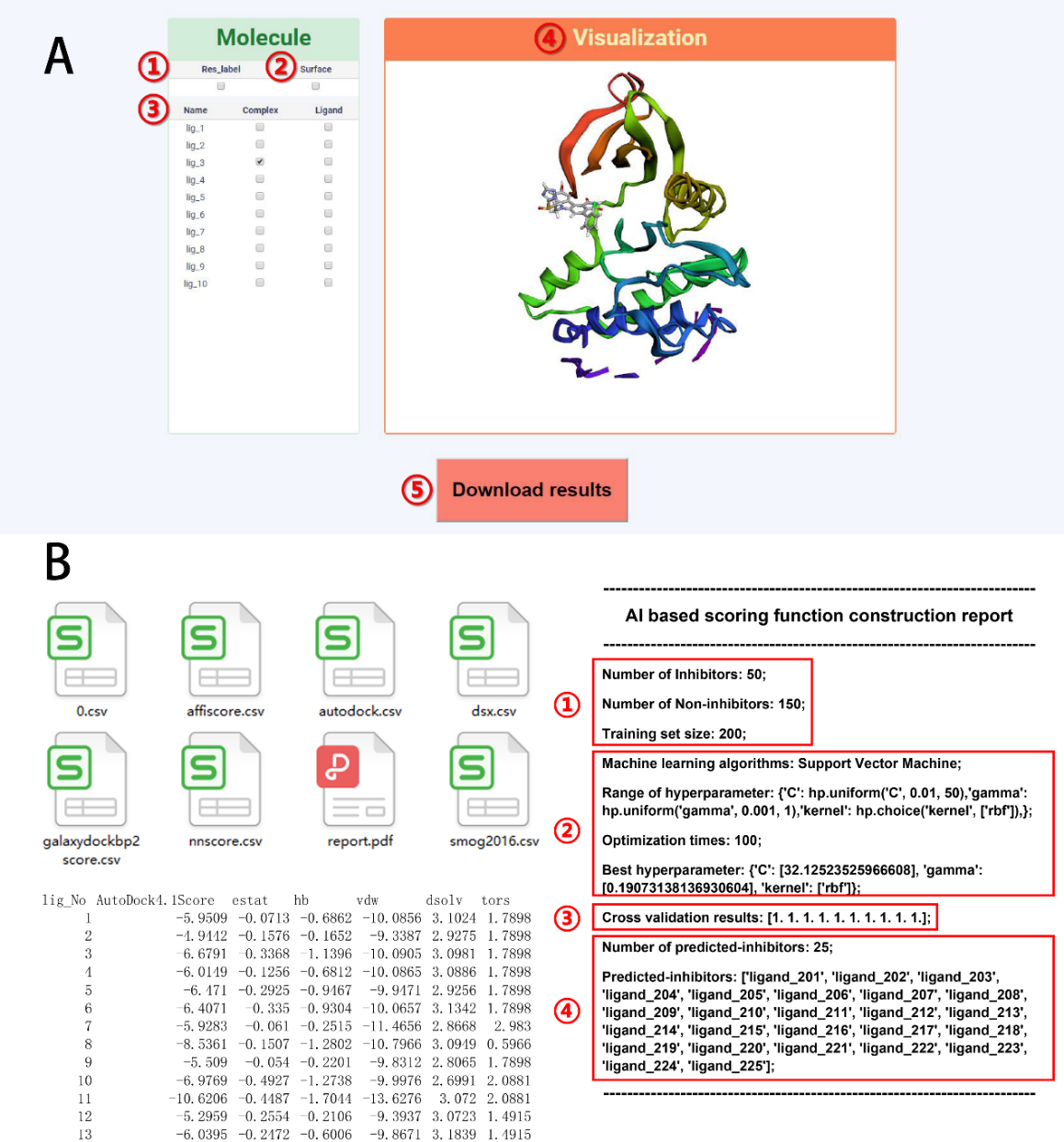
***

**Figure S5**. The result page and files of ASFP. (A) the result page mainly contains five parts (1) Res_label: if this checkbox is selected, the label of the protein residues will be added to the Visualization box, (2) Surface: select this checkbox to add a surface of the protein and the ligand, (3) Complex: if the checkbox is checked, the corresponding 3D model of the protein-ligand complex will be displayed in the Visualization box; Ligand: if the checkbox is checked, the corresponding 3D model of the ligand will show up in the Visualization box, (4) Visualization: the 3D model of the ligand or the protein-ligand complex will be shown, (5) Download results: click this button to get result files. (B) the results files are in the CSV format or in the PDF format. The CSV files contain different types of descriptors and the information stored in the PDF file can be divided into four parts: (1) information about the training set and information about the test set for online prediction module, (2) information about the ML algorithm and its hyperparameters, (3) model performance, and (4) the prediction results.

**REFERENCES**

1. Mysinger, M. M.; Carchia, M.; Irwin, J. J.; Shoichet, B. K., Directory of Useful Decoys, Enhanced (DUD-E): Better Ligands and Decoys for Better Benchmarking. *J. Med. Chem.* **2012**, 55, 6582-6594.

2. Wang, R. X.; Fang, X. L.; Lu, Y. P.; Yang, C. Y.; Wang, S. M., The PDBbind database: Methodologies and updates. *Journal of Medicinal Chemistry* **2005**, 48, 4111-4119.

3. Bergstra, J.; Yamins, D.; Cox, D. D., Making a Science of Model Search: Hyperparameter Optimization in Hundreds of Dimensions for Vision Architectures. *International Conference on Machine Learning* **2013**, 30, 115-123.

4. Li, J.; Abel, R.; Zhu, K.; Cao, Y.; Zhao, S.; Friesner, R. A., The VSGB 2.0 model: a next generation energy model for high resolution protein structure modeling. *Proteins* **2011**, 79, 2794-812.

5. Salam, N. K.; Adzhigirey, M.; Sherman, W.; Pearlman, D. A., Structure-based approach to the prediction of disulfide bonds in proteins. *Protein Engineering Design & Selection* **2014**, 27, 365-374.

6. Saunders, C.; Stitson, M. O.; Weston, J.; Holloway, R.; Bottou, L.; Scholkopf, B.; Smola, A., Support vector machine. *Computer Science* **2002**, 1, 1-28.

7. Breiman, L., Random Forests. *Machine Learning* **2001**, 45, 5-32.

8. Chen, T.; Guestrin, C., In *Proceedings of the 22nd ACM SIGKDD International Conference on Knowledge Discovery and Data Mining*; Association for Computing Machinery: San Francisco, California, USA, 2016, pp 785–794.

9. Ballester, P. J., Ultrafast shape recognition: method and applications. *Future Medicinal Chemistry* **2011**, 3, 65-78.

10. Verdonk, M. L.; Cole, J. C.; Hartshorn, M. J.; Murray, C. W.; Taylor, R. D., Improved protein-ligand docking using GOLD. *Proteins-Structure Function and Genetics* **2003**, 52, 609-623.

11. Wagener, M.; Sadowski, J.; Gasteiger, J., AUTOCORRELATION OF MOLECULAR-SURFACE PROPERTIES FOR MODELING CORTICOSTEROID-BINDING GLOBULIN AND CYTOSOLIC AH RECEPTOR ACTIVITY BY NEURAL NETWORKS. *Journal of the American Chemical Society* **1995**, 117, 7769-7775.

12. Eldridge, M. D.; Murray, C. W.; Auton, T. R.; Paolini, G. V.; Mee, R. P., Empirical scoring functions .1. The development of a fast empirical scoring function to estimate the binding affinity of ligands in receptor complexes. *Journal of Computer-Aided Molecular Design* **1997**, 11, 425-445.

13. Halgren, T. A.; Murphy, R. B.; Friesner, R. A.; Beard, H. S.; Frye, L. L.; Pollard, W. T.; Banks, J. L., Glide: A new approach for rapid, accurate docking and scoring. 2. Enrichment factors in database screening. *Journal of Medicinal Chemistry* **2004**, 47, 1750-1759.

14. Friesner, R. A.; Murphy, R. B.; Repasky, M. P.; Frye, L. L.; Greenwood, J. R.; Halgren, T. A.; Sanschagrin, P. C.; Mainz, D. T., Extra precision glide: Docking and scoring incorporating a model of hydrophobic enclosure for protein-ligand complexes. *Journal of Medicinal Chemistry* **2006**, 49, 6177-6196.

15. Zang, Q.; Rotroff, D. M.; Judson, R. S., Binary Classification of a Large Collection of Environmental Chemicals from Estrogen Receptor Assays by Quantitative Structure–Activity Relationship and Machine Learning Methods. *Journal of Chemical Information Modeling* **2013**, 53, 3244-3261.

16. Hou, T.; Li, N.; Li, Y.; Wang, W., Characterization of Domain–Peptide Interaction Interface: Prediction of SH3 Domain-Mediated Protein–Protein Interaction Network in Yeast by Generic Structure-Based Models. *Journal of Proteome Research* **2012**, 11, 2982-2995.

17. Li, N.; Hou, T.; Ding, B.; Wang, W., Characterization of PDZ domain‐peptide interaction interface based on energetic patterns. *Proteins: Structure* **2013**, 81, 1676-1676.

18. Li, X.; Chen, L.; Cheng, F.; Wu, Z.; Tang, Y., In Silico Prediction of Chemical Acute Oral Toxicity Using MultiClassification Methods. *Journal of Chemical Information Modeling* **2014**, 54, 1061-1069.

19. Sun, H.; Pan, P.; Tian, S.; Xu, L.; Kong, X.; Li, Y.; Dan Li; Hou, T., Constructing and Validating High-Performance MIEC-SVM Models in Virtual Screening for Kinases: A Better Way for Actives Discovery. *Sci Rep* **2016**, 6, 24817.

20. Rego, N.; Koes, D., 3Dmol.js: molecular visualization with WebGL. *Bioinformatics* **2015**, 31, 1322-1324.
